# Targeted mutagenesis in Anaplasma marginale to define virulence and vaccine development against bovine anaplasmosis

**DOI:** 10.1371/journal.ppat.1010540

**Published:** 2022-05-16

**Authors:** Paidashe Hove, Swetha Madesh, Arathy Nair, Deborah Jaworski, Huitao Liu, Jonathan Ferm, Michael D. Kleinhenz, Margaret A. Highland, Andrew K. Curtis, Johann F. Coetzee, Susan M. Noh, Ying Wang, Dominica Genda, Roman R. Ganta

**Affiliations:** 1 Center of Excellence for Vector-Borne Diseases (CEVBD), Department of Diagnostic Medicine/Pathobiology, Manhattan, Kansas, United States of America; 2 Department of Pathobiology, School of Veterinary Medicine, St. George’s University, West Indies, Grenada; 3 Department of Clinical Sciences, Kansas State University, Manhattan, Kansas, United States of America; 4 Department of Anatomy and Physiology and, College of Veterinary Medicine, Kansas State University, Manhattan, Kansas, United States of America; 5 Animal Diseases Research Unit, USDA-ARS, 3003 ADBF, Pullman, Washington, United States of America; Stanford University School of Medicine, UNITED STATES

## Abstract

Tick-borne *Anaplasma* species are obligate, intracellular, bacterial pathogens that cause important diseases globally in people, agricultural animals, and dogs. Targeted mutagenesis methods are yet to be developed to define genes essential for these pathogens. In addition, vaccines conferring protection against diseases caused by *Anaplasma* species are not available. Here, we describe a targeted mutagenesis method for deletion of the phage head-to-tail connector protein (*phtcp*) gene in *Anaplasma marginale*. The mutant did not cause disease and exhibited attenuated growth in its natural host (cattle). We then assessed its ability to confer protection against wild-type *A*. *marginale* infection challenge. Additionally, we compared vaccine protection with the mutant to that of whole cell *A*. *marginale* inactivated antigens as a vaccine (WCAV) candidate. Upon infection challenge, non-vaccinated control cattle developed severe disease, with an average 57% drop in packed cell volume (PCV) between days 26–31 post infection, an 11% peak in erythrocytic infection, and apparent anisocytosis. Conversely, following challenge, all animals receiving the live mutant did not develop clinical signs or anemia, or erythrocyte infection. In contrast, the WCAV vaccinees developed similar disease as the non-vaccinees following *A*. *marginale* infection, though the peak erythrocyte infection reduced to 6% and the PCV dropped 43%. This is the first study describing targeted mutagenesis and its application in determining *in vivo* virulence and vaccine development for an *Anaplasma* species pathogen. This study will pave the way for similar research in related *Anaplasma* pathogens impacting multiple hosts.

## Author summary

This is the first study describing targeted mutagenesis and its application in determining virulence and vaccine development for an *Anaplasma* species pathogen. The study is important in advancing similar research in related *Anaplasma* pathogens impacting multiple hosts. We demonstrated the successful development of a complete gene deletion mutation in *A*. *marginale*. Further, this *in vivo* infection study was performed in the natural host and established that growth of *A*. *marginale* is greatly limited when the *phtcp* gene is deleted. This study also demonstrated that cattle challenged with the virulent wild-type *A*. *marginale* four weeks after receiving the mutant *A*. *marginale* vaccine (MLAV) neither developed clinical disease nor did they exhibit hematologic abnormalities, such as anemia. On the contrary, non-vaccinated and WCAV vaccinated cattle challenged with virulent wild-type *A*. *marginale* developed clinical anaplasmosis with severe anemia, erythrocyte anisocytosis, and high-load infection in erythrocytes.

## Introduction

Over the last few decades, several new, emerging and reemerging tick-borne rickettsial diseases have been identified as a major public health concern in the USA and globally [1–5]. Similarly, tick-borne diseases are responsible for major economic losses in food animal production throughout the world [6,7]. The much-needed targeted mutagenesis methods are yet to be fully developed and are of great importance for defining genes essential for several obligate intracellular bacterial pathogens, including tick transmitted *Anaplasmataceae* pathogens [8–11]. Notably, the lack of targeted mutagenesis methods is regarded as a major obstacle for advancing research in defining the pathogenesis of obligate intracellular bacteria and developing effective interventions, including vaccines [11]. Mutagenesis methods can aid in developing efficacious vaccines against such pathogenic bacteria [11–15]. We recently reached a milestone in establishing targeted genetic manipulation methods by developing successful allelic exchange-based targeted mutagenesis in *Ehrlichia chaffeensis*, a member of the *Anaplasmataceae*; the method aided in creating mutations in several genes and supported both disruption and restoration of a gene function [14]. Similar research, however, is yet to be advanced in other related rickettsial pathogens, including the genera *Anaplasma* and *Neorickettsia*. We reasoned that the targeted mutagenesis methods we developed for *E*. *chaffeensis* could be adapted to other *Anaplasmataceae* pathogens, including *Anaplasma* species.

In our recent studies, the functional disruption in the gene encoding the membrane-bound phage head-to-tail connector protein (*phtcp*) of *E*. *chaffeensis* (gene tag # ECH_0660) causes rapid pathogen clearance from a host, while inducing sufficient immune response to confer protection against wild-type infection challenge by both intravenous inoculation and by tick transmission [16,17]. Our recent data also suggest that the functional disruption in the *E*. *chaffeensis phtcp* gene is likely detrimental in altering the pathogen’s ability to obtain metal ions to support its growth within a phagosome of infected host macrophages [18]. Since the *phtcp* gene orthologs are well-conserved in all known *Anaplasma* and *Ehrlichia* species [18], we reasoned that targeted mutagenesis disrupting the function of *phtcp* orthologs in other related *Anaplasmataceae* pathogens could facilitate establishing mutagenesis methods for application in developing live attenuated vaccines.

*Anaplasma marginale* infects bovine erythrocytes and causes diverse clinical signs, including persistent high fever, anemia, icterus, weight loss, abortion in pregnant cows, lowered milk production in dairy cattle, and reduced meat production in beef cattle. *A*. *marginale* is also reported to cause high mortalities in beef and dairy cattle globally [6,19,20]. Despite the estimated major global economic losses due to bovine anaplasmosis, amounting to billions of US dollars annually, efficacious and safe vaccines for worldwide use in controlling the disease are currently not available [6,21,22]. Although a heterologous live *A*. *centrale* blood vaccine is used as a means of reducing disease severity caused by *A*. *marginale* in parts of sub-Saharan Africa, Israel, Uruguay, and Australia, it is not in use in parts of the world, including Europe and North America as it may result in introducing other blood-borne pathogens [6,23]. Whole cell inactivated vaccines have been reported with limited efficacy [24,25]. While a similar experimental killed vaccine is marketed by the Louisiana State University Agricultural Center, there are no scientific reports describing its production and efficacy. Additionally, development of subunit vaccines against bovine anaplasmosis has had limited success [6,26–34]. A recent study reported the feasibility of developing a live attenuated vaccine using an *A*. *marginale* strain with a random insertion mutation, though the work has yet to progress beyond the initial description [35]. Given the insufficiencies of the current control options, coupled with the high economic burden associated with bovine anaplasmosis, investigations focused on developing alternative vaccines for control of this disease remain a high-priority goal.

In the current study, we report the development of a targeted mutagenesis method for an *Anaplasma* species; we deleted the *phtcp* gene from the *A*. *marginale* genome and demonstrated that cattle infected with this mutant (MLAV) exhibited no clinical disease and the mutant had an *in vivo* growth defect. Further, we present data showing that prior infection with the mutant offered protection against virulent disease caused by the wild-type *A*. *marginale* and kept erythrocyte infection below detectable levels, as assessed by light microscopy. We also tested an *A*. *marginale* whole cell antigen-based inactivated vaccine (WCAV) to compare its efficacy with that of the MLAV and report that it failed to prevent clinical disease following wild-type infection challenge. The current study represents the first description of targeted mutagenesis in an *Anaplasma* species and illustrates its application in defining a gene essential for *in vivo* bacterial growth, making advancement towards developing a live attenuated vaccine to protect cattle from severe anaplasmosis.

## Results

### 1. Construction of the homologous recombination cassette for use in the *A*. *marginale phtcp* gene deletion

**Fig 1:**
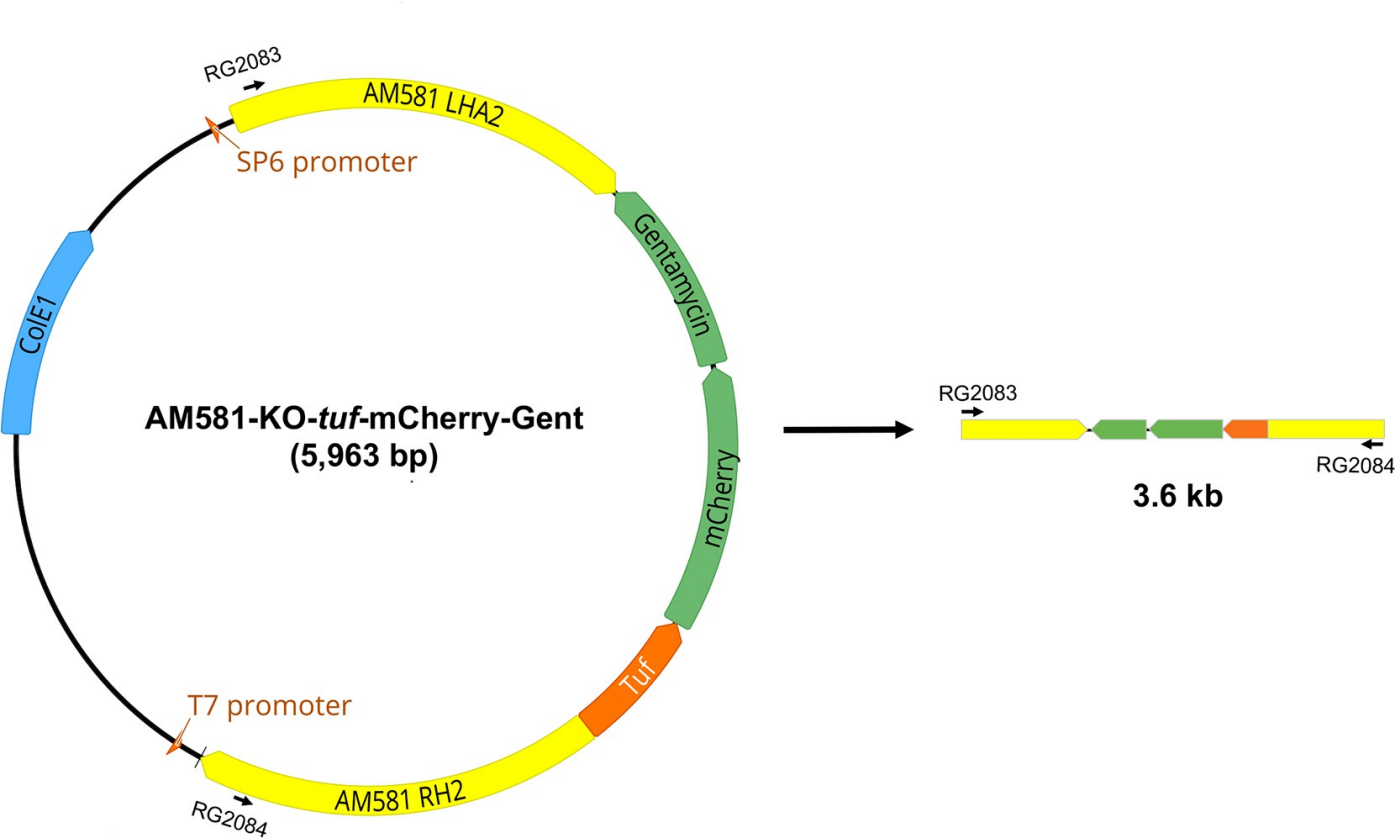
Plasmid map of AM581-KO-*tuf-*mCherry-Gent construct. Left and right homology arms for targeted allelic exchange are shown on the plasmid map. The segment containing the left and right homology arms separated by the *E*. *chaffeensis tuf* promoter segment to drive the expression of mCherry and gentamicin resistance cassette was amplified for use in allelic exchange homologous recombination.

Targeted mutagenesis methods that we reported previously for *E*. *chaffeensis* [14] were successfully adapted in this study to create a targeted deletion mutation in the *A*. *marginale* genome. To generate a gene deletion mutation, 1.1 kb each of *A*. *marginale* St. Maries strain (GenBank # CP000030) genomic DNA segments upstream and downstream to the *phtcp* gene (gene tag # AM581) were engineered to serve as the homology arms in the mutagenesis construct. The fragments were positioned upstream and downstream to mCherry and gentamicin resistance gene coding sequences to be transcribed from the *E*. *chaffeensis tuf* promoter [14]. The recombinant plasmid construct (AM581-KO-*tuf-*mCherry-Gent) was used for the homologous recombination experiments (Fig 1).

### 2. Establishing mutagenesis in *A*. *marginale* by homologous recombination

**Fig 2:**
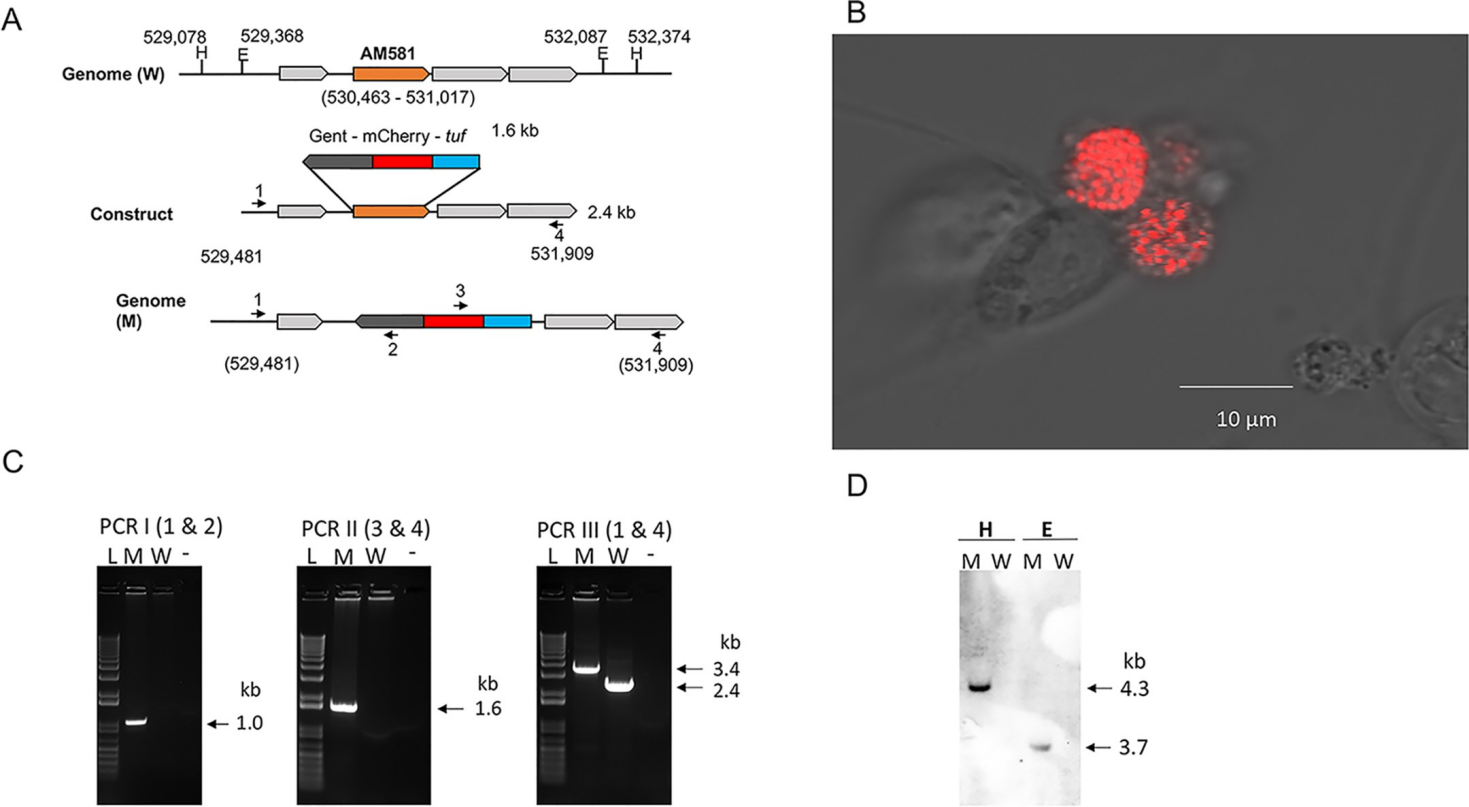
Targeted allelic exchange mutagenesis deleting *A*. *marginale* AM581 gene encoding *phtcp*. **A)** A cartoon illustrating the genomic region selected for preparing the allelic exchange construct and construct design. Restriction enzyme sites used in defining the mutation development [EcoRV (E) and HindIII (H)] are presented with their genomic coordinates. The size of the inserted fragment (*tuf*-mCherry-Gent) was included. The PCR primer sused in the analysis were identified as numbers 1 to 4. **B)** The AM581 gene deletion mutant in ISE6 tick cell culture expressing mCherry. The mCherry expression was detected by confocal microscopy using 40x magnification lenses. (The length bar on the bottom left corner of the figure to define the size; 10 microns). **C)** PCR analysis to define the mutation. Three different PCRs were performed: in PCR I, primers targeting the genomic region upstream to the insertion and the mCherry coding sequence were used; PCR II primers targeted to the mCherry coding region and downstream to the inserted region; in PCRIII, primers were targeted to *A*. *marginale* sequence upstream and downstream to the inserted fragment. (L, 1 kb plus molecular weight DNA markers; W, wild-type *A*. *marginale* genomic DNA; M, *phtcp* mutant genomic DNA). **D)** Southern blot analysis of genomic DNAs (W, wild-type and M, mutant) digested with EcoRV (E) and HindIII (H). The DNA blot analysis was performed using an mCherry gene segment as the probe.

The protocol for creating the targeted gene disruption mutation in *A*. *marginale* (St. Maries strain) was similar to the method we reported recently for *E*. *chaffeensis* [14] with a few minor modifications. The genomic region selected for preparing the allelic exchange construct, genomic coordinates, inserted segment, and selected restriction enzyme sites used in defining the mutant are depicted in Fig 2A. Gentamicin-resistant *A*. *marginale* cultures expressing mCherry were identified after several weeks of assessment in culture (Fig 2B). PCR assays targeting the upstream and downstream genomic regions used in the allelic exchange and to the inserted mCherry sequence confirmed the presence of the deletion mutation (Fig 2C). The AM581 gene deletion was further verified by a 3^rd^ PCR assay amplifying the targeted insertion region, which produced the expected larger amplicon from the mutant compared to that for wild-type (Fig 2C). Clonal purity of the mutant bacterium was further confirmed by Southern blot analysis following digestion of the mutant genomic DNA with HindIII or EcoRV restriction enzymes and hybridized with mCherry gene segment probe. Only the predicted restriction digested fragments were observed in the DNA recovered from the mutant, but not in the genomic DNA recovered from wild-type *A*. *marginale* (Fig 2D).

### 3. Development of a virulent bovine anaplasmosis infection model

Infection progression in an 18-month-old steer receiving blood stabilate infection was assessed for 92 days by observing for clinical signs, complete blood count (CBC) analysis, and in light microscopy examination for the presence of infection in erythrocytes. *A*. *marginale* erythrocytic inclusions were observed from day 8 post infection and peaked by day 29 to 25% of RBCs containing inclusions. The animal also had occasional spikes of temperature. The PCV and hemoglobin levels also steadily declined falling to below normal range by day 28 to 22.8% (normal range: 24–37%) and 7.1 g/dL (normal range: 8–15 g/dL), respectively (not shown). Blood was collected on alternate days when the bacteremia levels were about 11%, for use in preparing stabilates for infection experiments described below.

### 4. Impact of *A*. *marginale phtcp* gene disruption on *in vivo* infection in erythrocytes and clinical disease progression

To test the hypothesis that gene deletion in the *A*. *marginale phtcp* gene is detrimental for the pathogen’s *in vivo* growth, approximately 3 x 10^8^ mutant organisms each were intravenously (IV) inoculated into each steer. The steers were monitored daily for 28 days for clinical symptoms, changes in complete blood counts and for the presence of infected erythrocytes. The mutant-inoculated steers did not develop any clinical signs or anemia, and did not exhibit infected erythrocytes detectible by light microscopy. The animals also did not appear to have erythrocyte anisocytosis (not shown).

### 5. Assessment of attenuated mutant and whole cell inactivated antigens as vaccines

We then assessed if the prior inoculation of the *A*. *marginale* gene deletion mutant (as a modified live attenuated vaccine; MLAV) in cattle (n = 3) confers protection against disease progression resulting from virulent infection challenge. Similarly, we tested inactivated *A*. *marginale* whole cell antigens as another vaccine candidate (WCAV) (n = 3). The WCAV was injected twice subcutaneously, after combining with AddaVax oil-in-water emulsion as the adjuvant, on day 0 and day 21. The infection challenges with the virulent *A*. *marginale* St. Maries strain were performed on day 35 using the same source of blood stabilate for MLAV, WCAV, and non-vaccinated steers. The non-vaccinated infection control group steers (n = 3) didn’t receive either vaccine, however, they were inoculated with the adjuvant in a similar manner as the WCAV steers. All three animals in the infection control group and two from the WCAV group developed occasional spikes of fever and exhibited clinical signs such as lethargy, inappetence, pale mucous membranes, anemia, and jaundice, while the MLAV animals had no recognized clinical signs before or after infection challenge (Table 1).

**Table 1:** Clinical signs observed for all *A*. *marginale* vaccine study groups.

		Steer number	Clinical Signs Observed
**Treatment Group**	**Adjuvant Only**	4493	• Inappetence (D23, 24, 27 PC)
		4496	• Fever (104.6°F—D25 PC) • Inappetence (D27 PC)
		HH6	• Fever (104°F–D27 PC) • Lethargy & Inappetence (D28 –D30 PC) • Pale mucous membranes, Thin and watery blood, Jaundice onset (D29 PC) • Animal euthanized prior to study completion–condition was not improving
	**Whole Cell Antigen Vaccine**	4491	• Mild fever (103.2°F, D24)
		4502	• Appeared normal
		4505	• Mild fever (~103°F, D25 –D27) • Reduced appetite (D30, 31 PC)
	**Modified Live Vaccine**	DP324	• Appeared normal
		4506	• Appeared normal
		HH5	• Appeared normal

#### 5.1. Complete blood count (CBC) analysis and clinical disease

**Fig 3:**
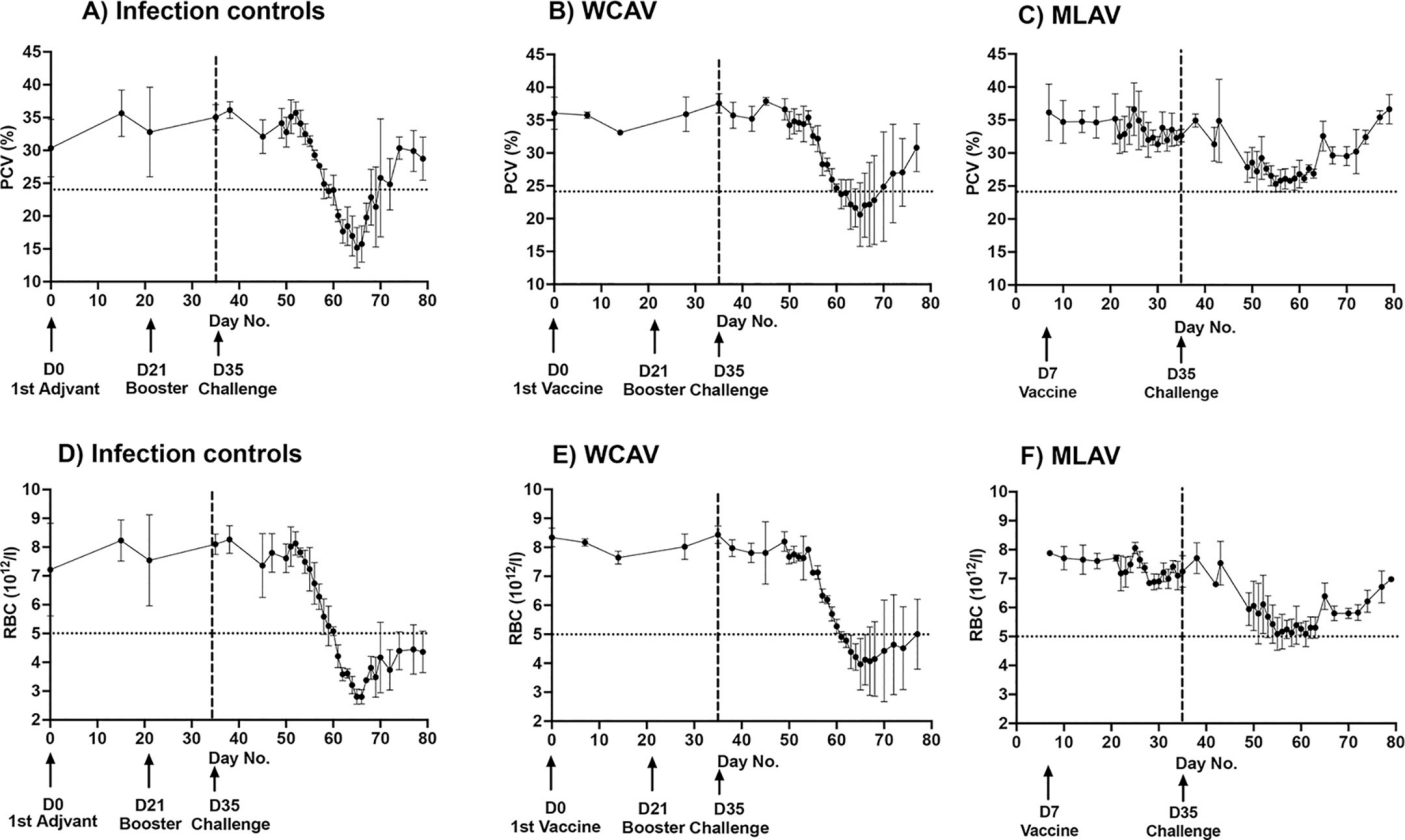
PCV and RBC count assessed in all three groups of steers. Average PCV and RBC values from animals sampled during the study period were presented for the non-vaccinated animals, WCAV and MLAV during vaccination phase and following infection challenge. Days of adjuvant injections (control group), vaccinations (WCAV and MLAV) and infection challenge day (all three groups) are identified in the figures. At significance level α = 0.05, One-way ANOVA with repeated measures showed significant differences for PCV (P = 0.0128*) and RBC (P = 0.0375*) levels for MLAV compared to infection controls or WCAV group animals for the days 60–70. However, no significant difference was observed for WCAV and infection control groups throughout the study period. Tukey’s multiple comparisons test also showed similar significant differences in PCV (P = 0.0056**) and RBC P = 0.0294*) for MLAV group animals compared to infection controls or WCAV animals between days 60 and 70. This method also did not yield significant difference between infection controls and WCAV animals.

In infection control animals, the PCV dropped to as low as 15% (a 57% drop) between days 26–31 post inoculation from 35% PCV observed in all groups of steers prior to infection challenges (Fig 3A). Disease severity in the infection control group warranted all three animals requiring close monitoring by the attending veterinarian, with one animal euthanized prior to study completion on day 31. The WCAV vaccinees also developed similar clinical disease as in non-vaccinated infection control group animals, while the MLAV vaccinees stayed healthy with no detectible clinical signs. The PCV in the WCAV animals also dropped below normal range to 20% (a 43% drop) (Fig 3B) with no significant difference noted between these two groups. In contrast, the MLAV animals had no notable variation in the PCV prior to infection challenge, while a transient mild drop in PCV (25%) was observed following wild-type infection challenge. The PCV values for MLAV were significantly higher than the non-vaccinated infected group animals for several days of assessment, spefically between days 20 and 35 post infection challenge (identified in Fig 3 as days 55–70). As with PCV, RBC counts also decreased below normal ranges for the infection controls and WCAV vaccinees, while the MLAV animals had significantly higher RBC counts and the values were within the normal range, as anticipated for healthy steers (Fig 3D–3F).

#### 5.2 Peripheral blood smear analysis by light microscopy

**Fig 4:**
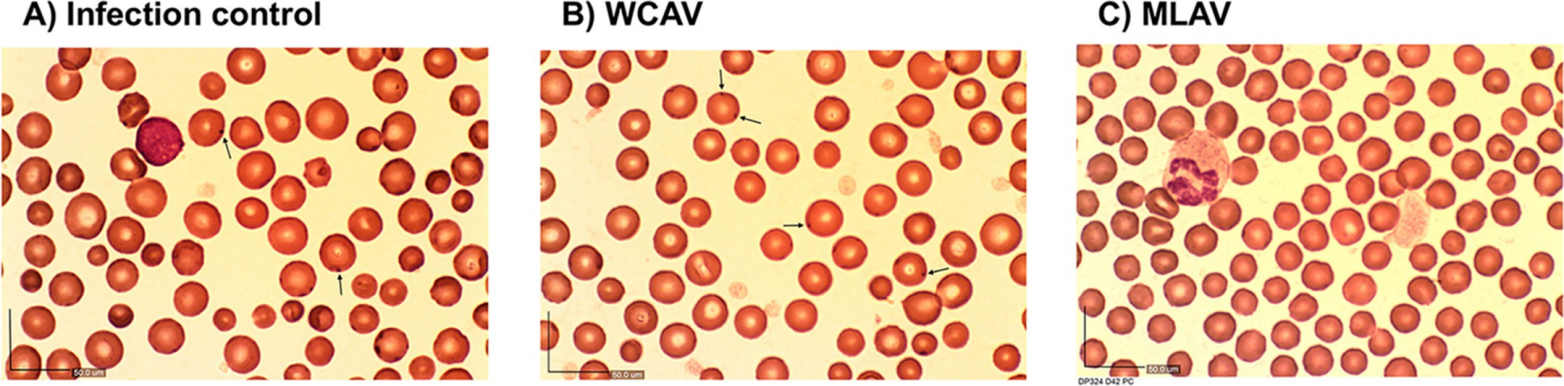
Thin blood smears assessed by light microscopy. Blood smear images were presented from one animal each for day 42 post infection challenge from all three experimental group animals. The images were collected following viewing with a 40x magnification. *A*. *marginale* inclusions are shown by black arrows (observed only in infection controls and WCAV animals; panels A and B), but not in MLAV animals (C). The *A*. *marginale* inclusions (identified with arrowhead lines) and anisocytosis are evident in both non-vaccinated infection controls WCAV vaccinees, but not in MLAV vaccinees. The length bars on the bottom left corner of each panel represent 50 microns.

**Fig 5:**
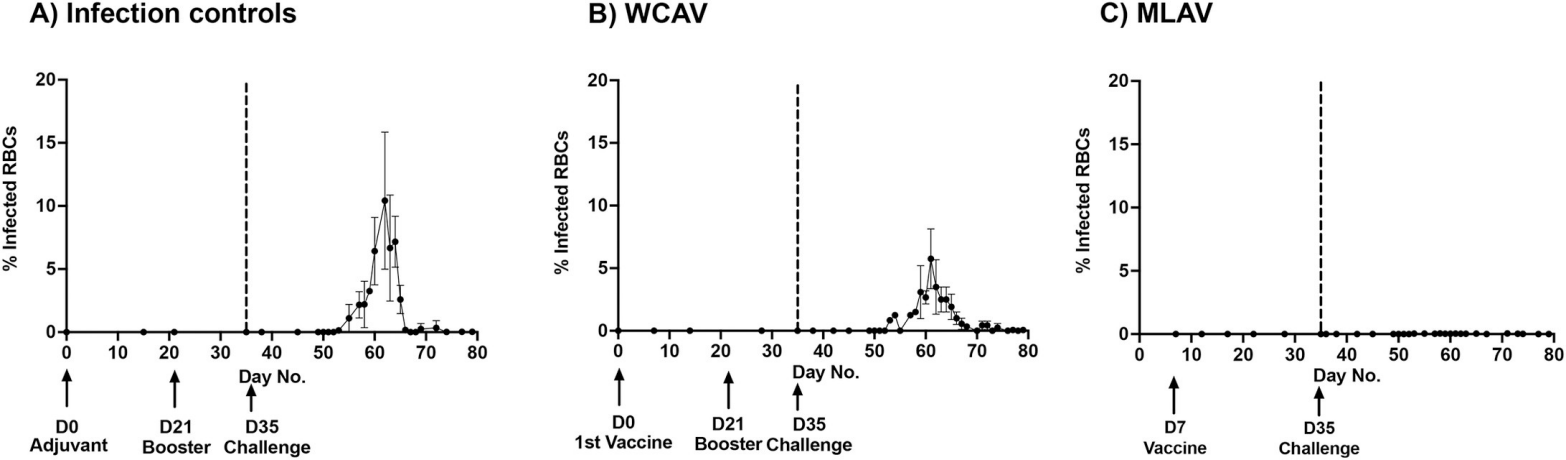
Percent infected erythrocytes as measured by light microscopy. Average percent infected erythrocytes following enumeration in 20 randomly chosen fields under 40x magnification and presented for all groups throughout the study period. Days of vaccinations and infection challenges were as in Fig 3. (significance for days 55–65) At significance level α = 0.05, One-way ANOVA with repeated measures showed that percent infected erythrocytes (bacteremia) were significantly different between the infection control, WCAV and MLAV groups (P = 0.0058**), for several days post-challenge. Additionally, Tukey’s multiple comparisons test showed significant differences between infection control and MLAV (P = 0.0334*) groups, as well as between WCAV and MLAV (P = 0.0117* groups. No significant difference between infection control and WCAV was observed.

Blood sampled over several weeks from animals in all three groups was assessed for the presence of *A*. *marginale* inclusions in erythrocytes by light microscopy (Figs 4 and 5); this assay is regarded as a gold standard for monitoring clinical bovine anaplasmosis [36–38]. Erythrocyte anisocytosis was evident in the blood smears of both the infection controls and WCAV vaccinees about four weeks after infection challenge, but not in the MLAV vaccinees (Fig 4). The mean percent of infected erythrocytes four weeks following challenge was >10% for the infection control group steers and declined thereafter (Fig 5A). Similarly, infected erythrocytes were observed in the WCAV vaccinees, although the peak infection was lower at about 6% (Fig 5B). In contrast, the MLAV vaccinees had no detectiable infected erythrocytes throughout the 80 days of assessment (Fig 5C).

#### 5.3. Systemic bacterial copy numbers assessed by quantitative PCR

**Fig 6:**
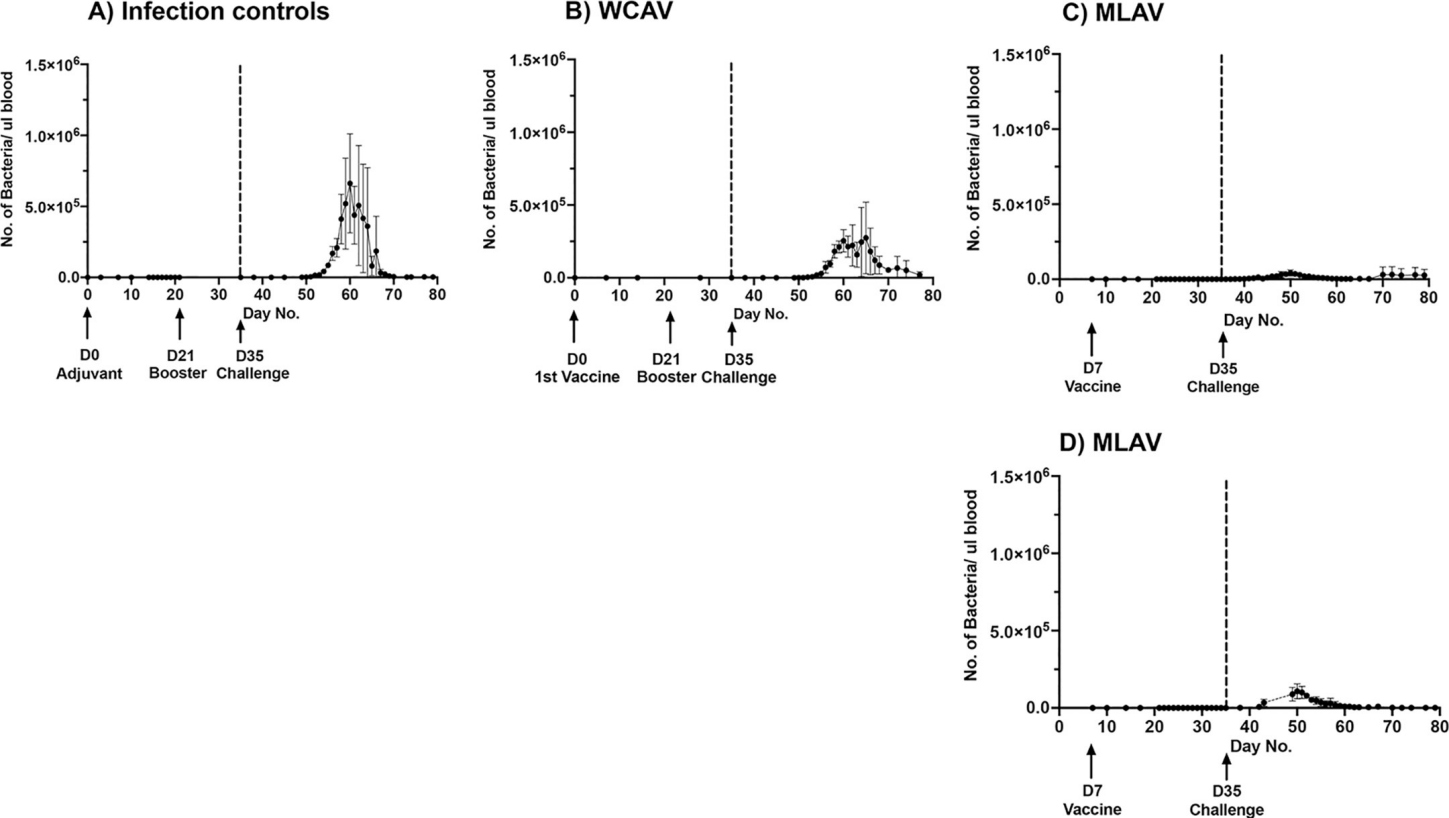
Bacterial numbers in blood assessed by real-time qPCR. The 16S rDNA TaqMan probe-based qPCR assays were performed for samples collected from all three groups animals over the study period (A-C). The MLAV group animal samples were also assessed by mCherry qPCR assay (D). Significance for days 55–65; at significance level α = 0.05, one-way ANOVA with repeated measures showed that 16S rDNA copy numbers of wild-type *A*. *marginale* bacteria were significantly different between the infection control, WCAV and MLAV groups (P = 0.0375*), for several days post-challenge. Additionally, Tukey’s multiple comparisons test showed a significant difference between infection control and MLAV groups (P = 0.0294*). No significant difference was observed between infection control and WCAV, or WCAV and MLAV.

We then assessed *A*. *marginale* DNA copies in blood by a more sensitive real-time quantitative PCR (qPCR) assay targeted to the 16S rDNA gene for the infection controls and WCAV vaccinees (Fig 6). For the MLAV vaccinees, qPCR was also performed targeting the mutant insertion segment; the mCherry gene (Fig 6D). The systemic bacterial loads, which peaked by 30 days post infection challenge, were higher for the infection control group than for the WCAV vaccinees, though the differences were not significant. The average estimated bacteria at peak were about 7 x 10^5^ per microliter of blood in non-vaccinated infection control group animals. In the WCAV vaccinees, the bacterial loads were also similarly observed but the peak bacterial numbers were two-thirds less (2.5 x 10^5^ per microliter of blood). The MLAV group animals had undetectable levels of mutant bacteria prior to virulent infection challenge, as determined by 16S rDNA qPCR and mCherry gene qPCR assays. Post infection, a significantly lower bacterial load was detected by qPCR assays in the MLAV vaccinees compared to WCAV and non-vaccinate infection control animals (Fig 6), with peak bacterial numbers detected as <10^5^ per microliter of blood in the MLAV animals. The differences between the MLAV group and the WCAV and infection control groups were most notable for days 55–65.

#### 5.4. Xenodiagnosis

**Fig 7:**
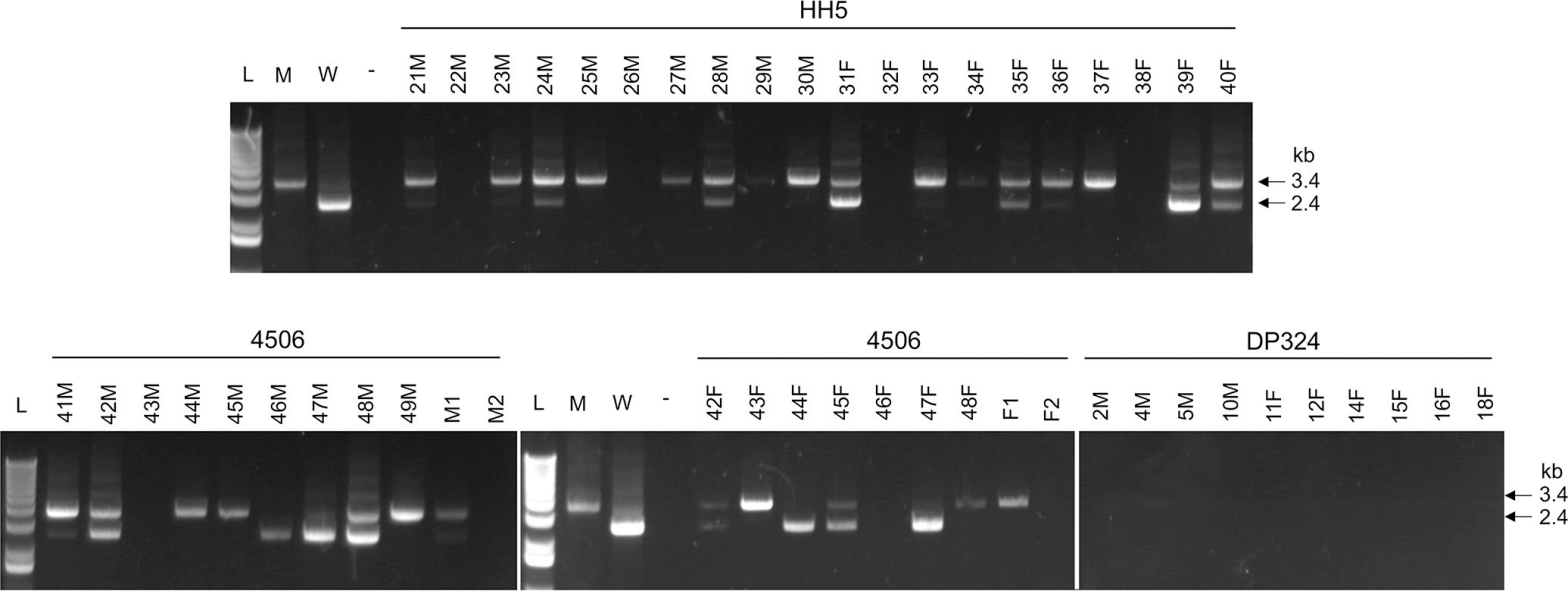
The presence of wild-type and mutant *A*. *marginale* in ticks assessed following blood feeding on MLAV group animals and their subsequent molting. Genomic DNAs from 20 ticks fed on each animal (10 males and 10 females) from steers 4506 and HH5 were tested for the presence of wild-type and *phtcp* mutant *A*. *marginale* by conventional PCR targeting to amplify the entire insertion-specific region; anticipated product size for wild-type and mutant are 2.4 kb and 3.4 kb, respectively. As a total of 60 randomly selected ticks (30 males and 30 females) fed on animal # DP324 were negative for both wild-type and mutant *A*. *marginale* by qPCR, 10 randomly selected ticks from this animal were also tested by conventional PCR. L, 1kb plus molecular weight markers; M, *A*. *marginale* mutant; W, Wild-type strain,—refers to no template containing negative control. The numbers with the letters M or F are to indicate the tick identification numbers and to indicate their sex; M, male and F, female.

To further assess the *A*. *marginale* infection status in animals post virulent infection challenges, a more sensitive xenodiagnosis assay was performed by allowing *D*. *variabilis* nymphal ticks to acquisition feed on all three groups of animals. Following blood feeding of nymphs and molting to adults, 20 randomly selected ticks (10 males and 10 females) per animal (60 total per group) were tested to determine the presence of *A*. *marginale* infection by performing the 16S rDNA gene qPCR. Fifty-nine out of 60 ticks from both the non-vaccinated infection group and the WCAV group animals were positive for *A*. *marginale*. Similar numbers of ticks from two MLAV group animals tested positive for two animals (animals #s, HH5 and 4506), while all 20 ticks from the third animal were negative for *A*. *marginale* infection (animal # DP324) (Table 2). To confirm the absence of *A*. *marginale* infection in the third MLAV vaccinee, 40 additional ticks were tested; all of which also tested negative for *A*. *marginale* infection (not shown). The Ct values are considerably higher for the ticks fed on MLAV animals compared to infection control group and WCAV group animals. Most ticks from the two MLAV vaccinees had Ct values within the 2 cycles when comparing between 16S rDNA and mCherry gene target qPCR values, while few ticks had Ct values greater than 2 cycles for the mCherry gene-specific pPCR compared to the 16S rDNA gene-specific qPCR, suggesting that these ticks harbored both mutant and wild-type bacteria. Thus, we re-evaluated tick DNAs derived from the MLAV vaccinees by a conventional PCR assay targeting the regions upstream to the mutation insertion region with expected size amplicons of 2.4 kb and 3.4 kb for the wild-type and mutant, respectively. We used 20 randomly selected ticks recovered from steers; #HH5 and #4506 and 10 ticks from steer #DP324 (Fig 7). Ticks recovered from animals HH5 and 4506 were positive primarily for the mutant-specific larger amplicon, while a few ticks were positive for the smaller wild-type *A*. *marginale-*specific amplicon. Some ticks were negative for both amplicons and similarly few ticks were positive only for the wild-type amplicon. Consistant with the qPCR assays, all 10 ticks from steer DP324 were negative for the conventional PCR assay.

**Table 2:** TaqMan qPCR data (Ct values) for ticks fed on steers.

Infection control group						
Animal ID	4493		4496		HH6	
	M	F	M	F	M	F
	21.5 (+)	19.9 (+)	15.5 (+)	16.8 (+)	16.4 (+)	14.5 (+)
	26.7 (+)	21.0 (+)	16.1 (+)	15.7 (+)	15.5 (+)	15.1 (+)
	21.5 (+)	20.1 (+)	18.0 (+)	13.9 (+)	16.3 (+)	21.5 (+)
	16.6 (+)	19.5 (+)	18.1 (+)	19.0 (+)	20.1 (+)	19.5 (+)
	-ve	21.2 (+)	14.8 (+)	18.7 (+)	18.3 (+)	17.5 (+)
	17.7 (+)	18.8 (+)	20.3 (+)	16.0 (+)	20.3 (+)	18.8 (+)
	23.6 (+)	18.2 (+)	17.3 (+)	16.6 (+)	16.8 (+)	17.7 (+)
	21.8 (+)	17.8 (+)	17.4 (+)	15.4 (+)	13.7 (+)	17.0 (+)
	18.6 (+)	21.0 (+)	17.5 (+)	16.6 (+)	18.4 (+)	13.7 (+)
	18.3 (+)	17.7 (+)	16.3 (+)	19.6 (+)	17.7 (+)	17.2 (+)
WCAV group						
Animal ID	4491		4502		4505	
	M	F	M	F	M	F
	22.9 (+)	21.2 (+)	34.8 (+)	22.4 (+)	22.8 (+)	23.6 (+)
	22.5 (+)	20.9 (+)	20.9 (+)	21.2 (+)	20.3 (+)	23.8 (+)
	24.7 (+)	21.2 (+)	22.6 (+)	20.2 (+)	21.4 (+)	22.9 (+)
	22.3 (+)	25.9 (+)	23.6 (+)	21.9 (+)	25.6 (+)	22.7 (+)
	20.5 (+)	24.9 (+)	21.3 (+)	23.3 (+)	23.7 (+)	21.8 (+)
	22.6 (+)	22.3 (+)	23.8 (+)	22.7 (+)	21.1 (+)	22.1 (+)
	22.2 (+)	22.8 (+)	21.7 (+)	20.1 (+)	24.0 (+)	20.8 (+)
	21.5 (+)	23.1 (+)	20.5 (+)	23.7 (+)	23.4 (+)	23.1 (+)
	25.4 (+)	20.5 (+)	26.5 (+)	21.9 (+)	21.0 (+)	21.9 (+)
	23.5 (+)	25.1 (+)	24.5 (+)	24.0 (+)	21.1 (+)	-ve
MLAV group						
Animal ID	16S rRNA		mCherry			
	M	F	M	F		
HH5	26.7 (+)	23.1 (+)	27.8 (+)	25.3 (+)		
	28.0 (+)	-ve	29.0 (+)	36.8 (+)		
	23.4 (+)	23.2 (+)	24.8 (+)	24.1 (+)		
	24.2 (+)	30.6 (+)	26.0 (+)	32.1 (+)		
	27.3 (+)	26.5 (+)	28.0 (+)	28.4 (+)		
	28.2 (+)	27.6 (+)	29.4 (+)	28.9 (+)		
	31.0 (+)	25.5 (+)	32.3 (+)	26.5 (+)		
	27.8 (+)	32.8 (+)	29.0 (+)	33.2 (+)		
	32.5 (+)	22.7 (+)	34.6 (+)	26.0 (+)		
	25.2 (+)	21.6 (+)	26.3 (+)	23.4 (+)		
4506	23.2 (+)	36.2 (+)	24.1 (+)	33.8 (+)		
	23.0 (+)	29.1 (+)	24.8 (+)	30.1 (+)		
	-ve	26.8 (+)	37.9 (+)	28.0 (+)		
	26.6 (+)	28.4 (+)	27.9 (+)	33.2 (+)		
	30.1 (+)	31.3 (+)	31.0 (+)	35.3 (+)		
	33.1 (+)	-ve	36.0 (+)	34.2 (+)		
	24.7 (+)	27.3 (+)	29.0 (+)	33.4 (+)		
	23.0 (+)	28.5 (+)	24.8 (+)	29.8 (+)		
	24.5 (+)	28.9 (+)	26.0 (+)	30.3 (+)		
	28.7 (+)	-ve	26.0 (+)	34.1 (+)		
DP324	-ve	-ve	-ve	-ve		
	-ve	-ve	-ve	-ve		
	-ve	-ve	-ve	-ve		
	-ve	-ve	-ve	-ve		
	-ve	-ve	-ve	-ve		
	-ve	-ve	-ve	-ve		
	-ve	-ve	-ve	-ve		
	-ve	-ve	-ve	-ve		
	-ve	-ve	-ve	-ve		
	-ve	-ve	-ve	-ve		

#### 5.5. Gross and histopathological analysis

**Fig 8:**
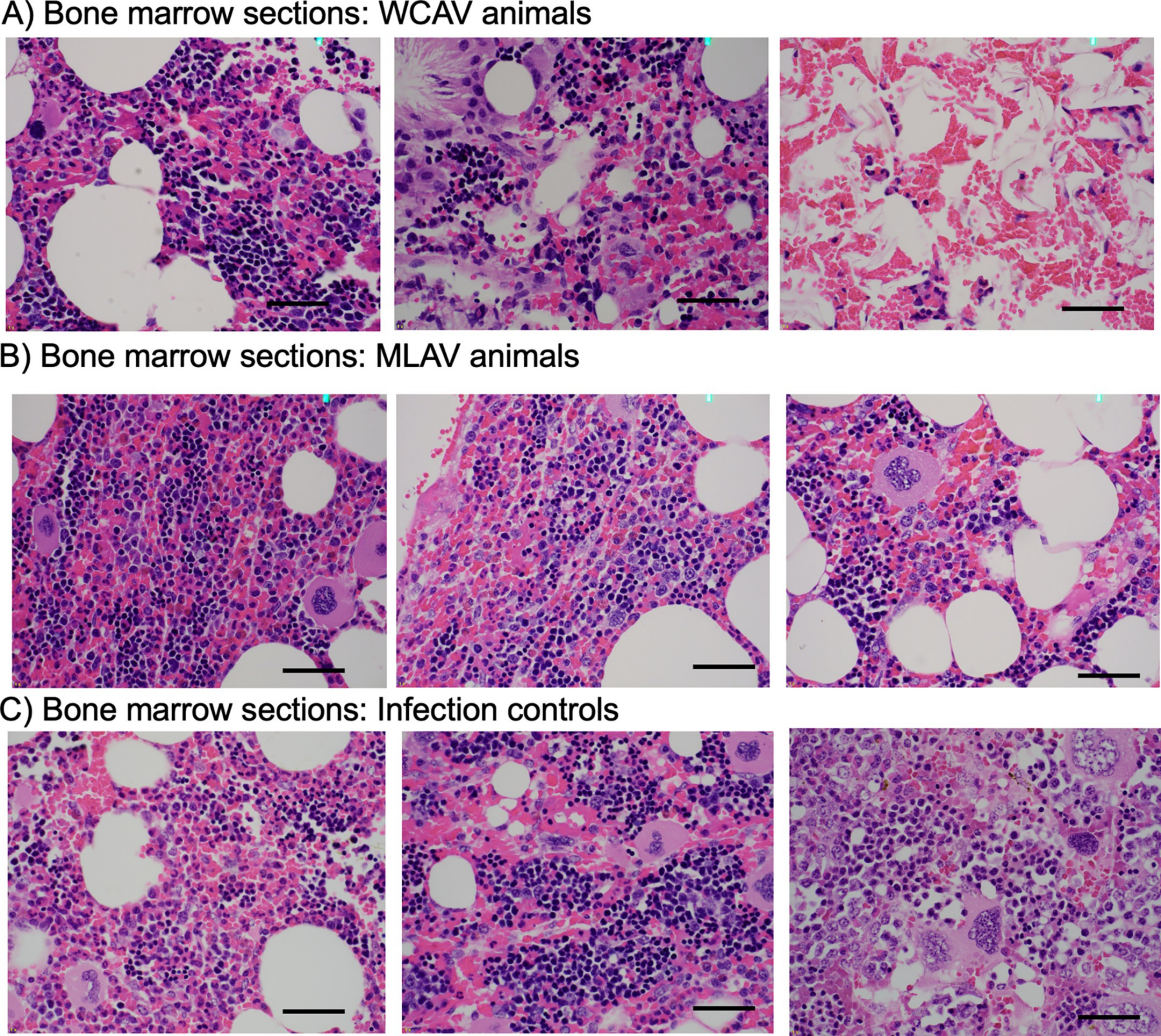
Post infection challenge histopathology sections of bone marrow at 40x magnification. From left to right panels: **A)** WCAV vaccinees (4491, 4502 and 4505); **B)** MLAV vaccinees (DP324, 4506 and HH5); **C)** Infection controls (4405, 4406 and HH6).

Animals in all three experimental groups had no lesions identified in visceral organs by gross or light microscopic examination. Histological examination of bone marrow from the WCAV vaccinees had fewer to no identifiable megakaryocytes, contained cholesterol clefting/atrophy of adipose, edema, and one animal lacked progenitor cells (Fig 8). Contrary to this, bone marrow from both non-vaccinated infection controls and MLAV vaccinees were histopathologically unremarkable (Fig 8).

#### 5.6. Total IgG expression assessed by enzyme-linked immunosorbent assays

**Fig 9:**
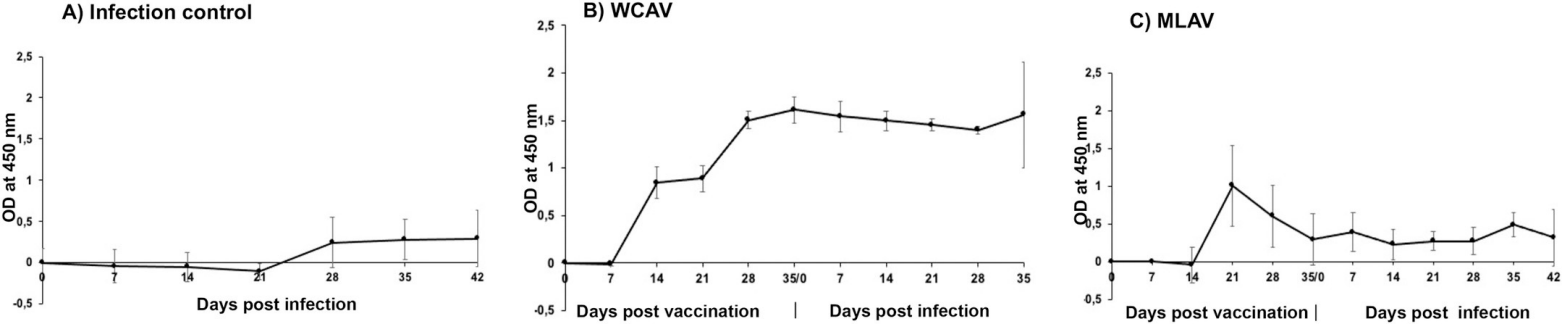
*A*. *marginale*-specific IgG response in cattle. Antigen-specific IgG antibodies were measured by ELISA in plasma samples collected from day zero (prior to infection) and multiple time points post vaccination and post challenge. Purified *A*. *marginale* whole cell antigens recovered from ISE6 cell cultures were used to coat the ELISA plates. Average absorbance values of plasma collected from steers within each group were plotted against plasma collection days. At, significance level α = 0.05, One-way ANOVA with repeated measures showed that IgG levels were significantly different between the infection control, WCAV and MLAV groups (P < 0.0001****), for several days post-challenge. Additionally, Tukey’s multiple comparisons test showed significant differences between infection control and WCAV groups (P = 0.0003***), infection control and MLAV groups (P = 0.0024**), and WCAV and MLAV groups (P = 0.0041**).

*A*. *marginale* whole cell antigen-specific IgG response was assessed by ELISA on plasma samples collected at multiple time points post vaccination and post challenge for the WCAV vaccinees and MLAV vaccinees, and post infection challenge for samples collected from non-vaccinated infection controls (Fig 9). *A*. *marginale*-specific IgG response was observed for both the WCAV and MLAV vaccinees, although the response in WCAV animals was greater and increased following booster vaccination; it was about three-fold higher compared to MLAV vaccinees. The IgG levels were significantly lower for the MLAV animal samples compared to WCAV. The non-vaccinated infection controls had the lowest IgG response.

## Discussion

Targeted mutagenesis in pathogenic bacteria having the ability to inactivate a gene and also to restore a gene function, including for pathogens of the order *Rickettsiales*, is a heavily sought after goal [11]. The order *Rickettsiales* has two families: *Rickettsiaceae* and *Anaplasmataceae*. To date, only two publications reported targeted mutations for *Rickettsia* species belonging to the *Rickettsiaceae* family; one targeted homologous recombination (allelic exchange) mutation in *Rickettsia prowazekii* reported in 2009 by Driskell *et al*. [39] and another targeted LtrA group II intron retrohoming system-based mutation in *Rickettsia rickettsii* in 2015 by Noriea *et al*. [9]. For the *Anaplasmataceae* family, we have previously reported the development of targeted mutagenesis by allelic exchsnge in creating mutations at few genomic sites in *Ehrlichia chaffeensis* [12,14].

The current study is the first in describing a targeted mutation in an *Anaplasma* species. The disruption mutation in the *E*. *chaffeensis* phage head to tail connector protein (*phtcp*) gene (gene tag # ECH_0660) has minimal impact for its *in vitro* growth, while inducing attenuated growth in two different vertebrate hosts [12,13]. As the *phtcp* gene orthologs are broadly conserved in *Anaplasma* and *Ehrlichia* species [18], we reasoned that it serves as an ideal target for defining targeted mutagenesis in diverse *Anaplasmataceae* organisms. Indeed, the current study demonstrated such feasibility in *A*. *marginale*. In this study, we demonstrated that the targeted mutagenesis methods we developed for *E*. *chaffeensis* [14] are applicable for mutagenesis experiments in both *Anaplasma* and *Ehrlichia* species. Considering the current success, it is likely that the allelic exchange mutagenesis can similarly be applied to other related *Anaplasmataceae* organisms, including the genera *Neorickettsia* and *Wolbachia* [2,5,40].

Previous studies involving *Anaplasma* species reported the use of transposon mutagenesis and it has remained the only option available for creating mutations [41–43]. The allelic exchange-based targeted mutagenesis will aid in defining genes essential for bacterial pathogenesis in a host, defining host-pathogen interactions, and developing prevention methods for diseases caused by several emerging tick-borne rickettsial diseases. Earlier, we reported that the *phtcp* gene disruption of *E*. *chaffeensis* causes an *in vivo* growth defect, possibly resulting from limiting the bacterial zinc and iron acquisition [18]. The data presented in the current study extends our prior data reporting that the functional *phtcp* protein is also critical for *A*. *marginale in vivo* growth. Previously, Munderloh’s group and Kocan’s group reported that cattle infected with *I*. *scapularis* tick cell culture-derived *A*. *marginale* develop the clinical disease similar to that observed when animals received blood stabilate infections [44,45]. The bacterial immunogenic surface proteins’ expression is also similar in *A*. *marginale* cultured in tick cell to that observed in infected erythrocytes [46]. Tick cell culture-derived *A*. *marginale* infection caused a significant drop in the PCV and a steady increase in bacteremia similar to cattle infected with blood stabilates. Contrary to these data, cattle infected with the *phtcp* gene deletion mutant resulted in the attenuated growth of *A*. *marginale* with no significant drop in PCV and rise in bacteremia [44,45]. While the possibility of attenuation of wild-type *A*. *marginale* during tick cell culture growth cannot be ruled out, the *phtcp* gene disruption mutation causing growth defect in *A*. *marginale* is consistent with our prior published data for *E*. *chaffeensis* having a similar gene mutation [12,16,17].

Bovine anaplasmosis continues to cause high economic losses throughout the world resulting from the reduced milk and meat production. Furthermore, the excessive use of tetracycline derivatives added as a food additive for reducing *A*. *marginale* infections also contributes to the economic burden and increases the antibiotic resistance risk to animals and humans [47,48]. Thus, a vaccine to prevent bovine anaplasmosis will be most valuable in both containing the disease and in reducing the antibiotic prophylactic used as a food additive. Our current study demonstrated that animals receiving one dose of the *phtcp* gene deletion mutant as a live vaccine offers the best protection in clearing the clinical disease, improving hematological parameters and also in reducing the systemic bacterial loads. Contrary to this, the WCAV vaccinees developed clinical disease similar to the non-vaccinated animals, although some improvements were noted in reducing both the bacterial infection in erythrocytes and anemia. Inadequate protection with WCAV against infection challenge is similar to a previous study by de la Fuente *et al*. [25]. This study prepared a whole cell inactivated antigen vaccine using *A*. *marginale* cultured in *I*. *scapularis* tick cell cultures. Over five decades ago, Brock *et al*. reported that vaccine-induced protection with inactivated bacterial antigens is not sufficient in protecting animals from bovine anaplassmosis [24].

*A*. *marginale* was undetectable in MLAV vaccinees in erythrocytes when assessed by light microscopy and lacked anemia. A more sensitive qPCR assay demonstrated the presence of both the mutant and wild-type *A*. *marginale* in the blood of MLAV vaccinees although the bacterial numbers were significantly lower compared to WCAV vaccinees and non-vaccinated animals. Further, xenodiagnosis substantiated the presence of low-level circulation of the mutant and wild-type *A*. *marginale*. The infection-persistence, however, was observed in only two of the three MLAV vaccinees. The data suggest that despite the absence of clinical disease and recovery from anemia, the MLAV did not offer complete sterile immunity at least in two of the three animals assessed.

The bone marrow was normal in MLAV vaccinees, thus the vaccine also helped to keep the bone marrow healthy as in comparison to non-vaccinated animals. It is unclear why WCAV vaccinees had the loss of megakaryocytes in bone marrow, and other changes, such as adipocyte atrophy, cholesterol clefts, and edema. One possible explanation is that the vaccine-induced immunity in WCAV vaccinees may have adversely impacted animals when receiving the virulent pathogen challenge. Modified live vaccines are likely to activate all arms of the immune system and provide immunity to combat clinical disease. We reported previously that *E*. *chaffeensis phtcp* gene mutant as the live vaccine provided complete protection for dogs against virulent pathogen infection challenge by IV inoculation and by tick transmission [16,17]. The current study assessed only IV infection challenge with a homologous virulent strain of *A*. *marginale*. Live *A*. *centrale* blood stabilate vaccine is generally regarded as having the ability to confer protection against *A*. *marginale* infections by both mechanical and tick-transmission challenge [6]. Thus, it is highly plausible that the *A*. *marginale phtcp* gene deletion mutant as a live vaccine offers sufficient protection against the disease resulting from diverse *A*. *marginale* strains transmitted from ticks and by mechanical transmission. This hypothesis, however, remains to be tested. Induction of T cell responses during intracellular bacterial infections is known to play a greater role in generating protection against infection than B cell responses [49,50]. Consistent with the previous observations [51], higher antibody response observed in the WCAV vaccinates did not aid in preventing the clinical disease, neither in reducing infection in erythrocytes nor in restoring the loss of erythrocytes. Protective response against bovine anaplasmosis, therefore, is more than just the induction of the B cell response; the present study is the critical first step in furthering studies to define the immune mechanisms of protection. The study is also important in determining if MLAV offers protection against diverse *A*. *marginale* strains transmitted mechanically or from an infected tick.

## Materials and methods

### Ethics statement

All experiments with cattle were performed in accordance with the Public Health Service (PHS) Policy on the Humane Care and Use of Laboratory Animals (https://olaw.nih.gov/policies-laws/phs-policy.htm), the U.S. Department of Agriculture’s (USDA) Animal Welfare Act & Regulations, and with the prior approval of the Kansas State University’s Institutional Animal Care and Use Committee (IACUC). At the conclusion of the study, all animals were euthanized according to the institutional IACUC recommendations, which are consistent with the recommendations of the Panel on Euthanasia of the American Veterinary Medical Association.

### *In vitro* cultivation of *A. marginale*

Both the wild-type and the *phtcp* mutant *A*. *marginale* St. Maries strain were propagated in *Ixodes scapularis* cell line culture (ISE6) at 34°C in the absence of CO_2_ as described earlier [41], except the media for culturing the mutant included gentamicin at a final concentration of 60 μg/ml.

### Generation of AM581 deletion construct

Homology arms of 1.1 kb each from both 5’ and 3’ to the *phtcp* gene (gene tag # AM581) of *A*. *marginale* St. Maries strain (GenBank #: CP000030) were amplified with the PCR primer sets listed in Table 3 and using the bacterial genomic DNA as the template. The *A*. *marginale* PCR products and a previously generated plasmid construct containing the contiguous *E*. *chaffeensis tuf* promoter, ORFs of mCherry, and the gentamicin resistance gene (tuf-mCherry-Gent) [14] were cloned into the pGGA plasmid vector (New England Biolabs, Ipswich, USA). The Golden Gate Assembly kit was used to assemble the fragments into the pGGA plasmid in the following order: 5’ *A*. *marginale* homology arm (1.1 kb), *tuf-*mCherry-Gent segment (1.6 kb), and 3’ *A*. *marginale* homology arm (1.1 kb). The final assembled recombinant plasmid is referred to as ‘AM581-KO-*tuf-*mCherry-Gent’. Standard molecular cloning protocols were followed to recover the recombinant plasmid that was transformed into the DH5α strain of *E*. *coli*. The integrity of the plasmid DNA, purified from the transformed *E*. *coli*, was verified by Sanger’s DNA sequencing analysis using the commercially available T7 and SP6 promoter primers (Integrated DNA Technologies, Coralville, IA, USA) annealing to the pGGA plasmid backbone. The recombinant plasmid was used as the template in a PCR to amplify the fragment containing the 5’ *A*. *marginale* homology arm, the *tuf-*mCherry-Gent segment, and the 3’ *A*. *marginale* homology arm (primers listed in Table 3). The PCR products were then purified as reported previously [14].

**Table 3:** List of oligonucleotides used in this study.

Name	Sequence (5’– 3’)	Target/Purpose
RG2073	ATCGGTGGTCTCCGGAGTTTCGCTATACAGAGCAGAA	AM581 Left Homology Arm
RG2056	ATCGGTGGTCTCCAACTAAACCACAGTGAAATTTTTAAGA	
RG2059	ATCGGTGGTCTCGTTGTGAACATTGCAGACCTG	AM581 Right Homology Arm
RG2074	ATCGGTGGTCTCGATGGATATCGGCCCTTGCTGTC	
RG2057	ATCGGTGGTCTCCAGTTTATGTTGCTGTACTTGGATC	Tuf-mCherry-Gentamycin
RG2058	ATCGGTGGTCTCCACAAAAATGTGACTATTAATTTTGACTTTT	
RG94	AAGCAAATGCTTTAGGTGCAT*	PCR I
RG2083	TCGGAGGTAGCGTGTCCTTA	
RG97	TCCGCAGGATGTTTCACATA*	PCR II
RG2084	GCATGGGCGTGGGTTTTTAG	
RG2083	TCGGAGGTAGCGTGTCCTTA	PCR III
RG2084	GCATGGGCGTGGGTTTTTAG	
RG2151	CTCAGAACGAACGCTGG	16S rDNA qPCR
RG2152	CATTTCTAGTGGCTATCCC	
RG2152P	FAM/CGCAGCTTG/ZEN/CTGCGTGTATGGT/IABkFQ	16S rDNA qPCR probe
RG2177	CGCGTGGGATATTCTTTC	mCherry qPCR
RG2178	CCGGGAAAGACAGTTTAAG	
RG2179	FAM/AGCCTATGT/ZEN/GAAACATCCTGCGGA/IABkFQ	mCherry qPCR probe
RG2161	ACAATCTCTCGGCAGGCAAA	AM581 (*phtcp*) gene (internal)
RG2162	CGGTCATGGAATCTCGCCTT	

*Reported in Wang et al. (2017) [15].

### Generation, clonal purification, verification, and propagation of *A*. *marginale phtcp* mutant

Approximately 20 μg of the above purified amplicon from the AM581-KO-*tuf-*mCherry-Gent plasmid were electroporated into ISE6 tick cell culture-derived *A*. *marginale* St. Maries organisms (~3 x 10^8^), by following our previously described method [14]. The electroporated bacteria were transferred to a cell suspension containing approximately 1 x 10^6^ uninfected ISE6 tick cells and propagated at 34°C in a T25 culture flask containing tick cell media for 24 h and then supplemented with 60 μg/ml final concentration of gentamicin. The cultures were maintained in the media with media changes once a week for the first three weeks and twice a week thereafter. The presence of mutant *A*. *marginale* expressing mCherry in cultures was monitored by fluorescence microscopy, while being maintained for several weeks in the presence of gentamicin to clear all wild-type bacteria.

To confirm the clonal purity of the mutant, three different PCR assays were performed using genomic DNA recovered from the mutant cultures; 1) forward primer specific to the inserted gentamicin gene segment and reverse primer targeted to the upstream genomic region 2) forward primer targeted to the downstream genomic region and reverse primer specific to the inserted mCherry gene segment, and 3) and forward and reverse primers targeted to the genomic regions upstream and downstream to the gene deletion-insertion mutation region (all primers were listed in Table 3). The PCR assays were performed in 25 μl reactions in 1x Q5 reaction buffer containing 2 mM MgCl_2_, 0.5 mM of each dNTP, 0.2 μM of each forward and reverse primers, 1 unit of Q5 *Taq* polymerase (New England Biolabs, Ipswich, MA, USA), and genomic DNA from wild-type or mutant organisms as the templates. The PCR cycling conditions for the first two PCRs were 98°C for 30 s, followed by 35 cycles of 98°C for 10 s, 65°C for 30 s, and 72°C for 2 min 30 s, then 72°C for 3 mins and a final hold at 10°C. For the third PCR assay, the annealing temperature was changed to 70°C. The PCR products were resolved on a 1.5% agarose gel containing ethidium bromide and visualized using a UV transilluminator. Clonal purity of the mutant was further assessed by Southern blot analysis using the mutant culture-derived genomic DNA digested with HindIII or EcoRV restriction enzymes and genomic DNA from wild-type *A*. *marginale* was similarly digested and used to serve as the control. The insertion-specific mCherry gene segment-specific DNA probe was used for detecting approximately 4.3 kb and 3.7 kb DNA fragments, respectively, only in genomic DNA recovered from the mutant cultures [12,13].

### *A*. *marginale* WCAV preparation

Wild-type *A*. *marginale* St. Maries strain was cultured in tick cell line to ~80% infection in 35 ml culture volume. The cultures were harvested and centrifuged 2,860 x g for 15 min at 4°C. The pellet was resuspended in 3 ml of 1 x PBS, passed through a 27 g bent needle for 10 times. The contents were centriguged at 500 x g for 3 min and the supernatant was collected and passed through a two micron filter. The filtrate was then centrifuged at 18,000 x g for 5 min, supernatant was removed and the pellet was resuspended in 1.25 ml of 1 x PBS. The recovered organisms were heat inactivated at 60°C for 30 min. The protein concentration was estimated by the BCA protein estimation method (ThermoFisher Scientific, Carlsbad, CA, USA). Approximately 200 μg of WCA per 1 ml 1X PBS was mixed with an equal volume of oil-in-water nano-emulsion suspension adjuvant, AddaVax (Invivogen, San Diego, CA, USA), for use as subcutaneously administered vaccine (WCAV). AddaVax is widely used in vaccine studies promoting the vaccine-induced immunity and without causing adjuvant-associated adverse reactions [52,53]. It is known for inducing Th1 response which is essential for the defense against intracellular parasites.

### Cattle infection and vaccine studies

Ten Holstein steers, approximately 18 months old, were obtained from an area in North Dakota reported to be free of bovine anaplasmosis (animal numbers were listed in Tables 1 and 2). To confirm no prior exposure, serum and whole blood from each animal were screened by an MSP5-based cELISA (*Anaplasma* Antibody Test Kit, cELISA v2;VMRD, Pullman, WA, USA) [54] and *A*. *marginale* 16S rDNA qPCR [55], respectively. The steers were housed at a vector-free animal facility at Kansas State University with food and water provided *ad libitum*. Steers could interact and socialize within their respective group animals. Animals were individually housed when tick studies were performed. Adequate space was also given to allow regular exercise/activity.

### Infection experiments in steers

Infection experiments were performed using either the *in vitro* cultured mutant organisms or the wild-type *A*. *marginale* St. Maries wild-type strain blood stabilates. For mutant *A*. *marginale* infection experiments, steers received ~3 x 10^8^ ISE6 tick cell culture-derived mutant organisms resuspended in 2 ml of 1X PBS. The infection challenges with wild-type St. Maries strain were performed IV using 2 ml each of blood stabilate (~6 x 10^9^ bacteria) (originating from the same batch) as per the previously described protocol [35]. The MLAV vaccinees were challenged with the virulent strain on day 28, while the WCAV group animals were challenged on day 35. Non-vaccinated infection control group steers received AddaVax adjuvant diluted in 1 x PBS (1:1) during the WCAV vaccination days. Prior to infection, blood stabilates were mixed with 5 ml freshly collected homologous blood plasma. Animals in MLAV, WCAV, and non-vaccinated groups received the same batch of inoculum.

### Animal monitoring, CBC, and assessment of systemic *A*. *marginale*

All cattle used in the current study were monitored daily for health and behavioral changes and twice weekly for body temperature. Veterinary care for the animals was overseen by a Kansas State University veterinarian. Throughout the study, 20 ml of blood was collected in EDTA tubes each week from all animals for plasma analysis. About 2 ml of blood was similarly collected twice per week for CBC analysis, performed on a VetScan HM5 Hematology Analyzer v2.3 (Zoetis, Union City, CA, USA). A small fraction of blood also in EDTA tubes was collected every other day to prepare blood smearls for light microscopic analysis of blood smears to monitor for erythrocyte *A*. *marginale* inclusions. Blood sampled from all animals were also assessed once per week for the presence of *A*. *marginale* by 16S rDNA PCR analysis. Additionally, two ml blood samples were collected daily throughout the acute phase of infection. All blood samples were processed either immediately or stored at 4°C for a maximum of 24 h prior to performing the described analyses. DNeasy Blood and Tissue DNA isolation kit (Qiagen, Germantown, MD, USA) was used to extract total genomic DNA from a 100 μl aliquot of the whole blood samples. Extracted genomic DNA from each sample was eluted in 150 μl of elution buffer. To assess *A*. *marginale* infection status, TaqMan probe-based qPCR assays were performed targeting the 16S rDNA as in [56]. Animals receiving the mutant *A*. *marginale* strain were also tested for the mutant-specific qPCR assay targeting the mCherry gene. The assay was standardized using the primers and TaqMan probes listed in Table 3. The qPCR assays were performed in 25 μl reactions with final concentrations of 1X reaction buffer containing 0.4 mM of each dNTP, 2.4 mM MgSO_4_, 0.1 μM concentration of both forward and reverse primers and the TaqMan probes, 1 unit of Platinum *Taq* polymerase (ThermoFisher Scientific, Carlsbad, CA, USA), and by including 2 μl each of genomic DNA as a template. Genomic DNA extracted from the wild-type *A*. *marginale* St. Maries strain was included as the positive control for the 16S rDNA assays, while DNA from the mutant *A*. *marginale* was used as the positive control for assays targeting the mCherry gene. Negative controls included all reactants and PCR-grade water in place of DNA template. The qPCR cycling conditions for the assays were 95°C for 3 mins, followed by 45 cycles of 94°C for 15 s, 50°C for 30 s and 60°C for 1 min (signal acquisition stage). Serial dilutions of the 16S rDNA gene- and mCherry gene-containing plasmids were used in the assays to define the copy numbers of molecules in the respective test samples. The Ct values obtained by fluorescence signal detection of the serially diluted plasmid controls ranging from 10^9^ to 10^1^ copies were used for generating standard curves and all assays were performed in triplicate.

### Xenodiagnosis of *A. marginale* by *Dermacentor variabilis*

Approximately 250 *D*. *variabilis* nymphal stage ticks were placed on all animals on day 19 post *A*. *marginale* wild-type infection challenge. Ticks were allowed to feed to repletion (~10 days) [57]. Fed ticks were carefully collected from the tick attachment cells and transferred to a humidified incubator with 14 h day light and 10 h darkness for molting to the adult stage, which took approximately 30 days. Genomic DNAs from molted ticks (equal numbers of males and females) fed on each animal were initially isolated and subjected to qPCR targeting to *A*. *marginale* 16S rDNA. The mCherry gene qPCR assays were also performed on DNAs recovered from ticks fed on the MLAV animals. Genomic DNA extractions were performed using a Qiagen DNeasy Blood and Tissue Kit (Qiagen, Germantown, MD, USA). The purified genomic DNA from each tick was recovered in 150 μl each of the elution buffer and stored at -20°C until use. A conventional PCR assay was also performed on tick DNAs using the forward and reverse primers targeted to the genomic regions upstream and downstream to the homology arm segments used in the mutagenesis experiment (primers listed in Table 3)

### Assessment for the presence of *A*. *marginale-*specific IgG production in steers by ELISA

Ninety-six well ELISA plates were coated with 10 μg/ml of host-cell free *A*. *marginale* total proteins prepared from the ISE6 tick cell-cultured organisms by incubating overnight at 4°C. The wells were blocked with the blocking buffer (1X PBS containing 1% BSA) and incubated at 37°C for 1 h. Plasma samples from the animals were diluted 1:200 in the blocking buffer, added to wells and incubated at 37°C for 1 h. The plates were then washed three times with wash buffer (1X PBS containing 0.05% Tween 20). Finally, the HRP conjugated anti-bovine IgG (Invitrogen, Frederick, MD, USA) at 1:2,000 dilution was added to the wells and incubated at 37°C for 1 h. The ELISA plates were washed three times with the wash buffer and then TMB substrate (EMD Millipore Corporation, Temecula, CA, USA) was added. After observing color development in the wells, the reactions were stopped by adding 0.1 M phosphoric acid solution (stop solution) and the absorbance at 450 nm was measured using an ELISA reader (Biotek Instruments, Winooski, VT, USA). All assays were performed in triplicate and the mean absorbance values and standard deviations were calculated.

### Statistical analysis

One-way ANOVA with repeated measures and Tukey’s multiple comparisons tests were performed using GraphPad Software (La Jolla, CA, USA) at significance level, α = 0.05, to assess the differences in wild-type bacteremia (as assessed by 16S qPCR and light microscopy examination of peripheral thin blood smears), PCV, RBC and IgG levels between the three groups at each time point following challenge.
